# Is TNF alpha a mediator in the co-existence of malaria and type 2 diabetes in a malaria endemic population?

**DOI:** 10.3389/fimmu.2023.1028303

**Published:** 2023-05-05

**Authors:** Subulade A. Ademola, Oluwayemi J. Bamikole, Olukemi K. Amodu

**Affiliations:** Molecular Genetics Unit, Institute of Child Health, College of Medicine, University of Ibadan, Ibadan, Nigeria

**Keywords:** malaria, type 2 diabetes (T2D), TNF-α, malaria-endemic population, inflammation, cytokine, hypothesis, TCF7L2 gene

## Abstract

Malaria remains a disease of public health importance globally, especially in sub-Saharan Africa. Malaria deaths reduced globally steadily between 2000-2019, however there was a 10% increase in 2020 due to disruptions in medical service during the COVID-19 pandemic. Globally, about 96% of malaria deaths occurred in 29 countries; out of which, four countries (Nigeria, the Democratic Republic of the Congo, the Niger, and the United Republic of Tanzania) accounted for just over half of the malaria deaths. Nigeria leads the four countries with the highest malaria deaths (accounting for 31% globally). Parallelly, sub-Saharan Africa is faced with a rise in the incidence of Type 2 diabetes (T2D). Until recently, T2D was a disease of adulthood and old age. However, this is changing as T2D in children and adolescents is becoming an increasingly important public health problem. Nigeria has been reported to have the highest burden of diabetes in Africa with a prevalence of 5.77% in the country. Several studies conducted in the last decade investigating the interaction between malaria and T2D in developing countries have led to the emergence of the intra-uterine hypothesis. The hypothesis has arisen as a possible explanation for the rise of T2D in malaria endemic areas; malaria in pregnancy could lead to intra-uterine stress which could contribute to low birth weight and may be a potential cause of T2D later in life. Hence, previous, and continuous exposure to malaria infection leads to a higher risk of T2D. Current and emerging evidence suggests that an inflammation-mediated link exists between malaria and eventual T2D emergence. The inflammatory process thus, is an important link for the co-existence of malaria and T2D because these two diseases are inflammatory-related. A key feature of T2D is systemic inflammation, characterized by the upregulation of inflammatory cytokines such as tumor necrosis factor alpha (TNF-α) which leads to impaired insulin signaling. Malaria infection is an inflammatory disease in which TNF-α also plays a major role. TNF-α plays an important role in the pathogenesis and development of malaria and T2D. We therefore hypothesize that TNF-α is an important link in the increasing co-existence of T2D.

## Introduction: the hypothesis

Type 2 diabetes is a non-communicable disease of serious public health importance globally. It is the most common type of diabetes mellitus, accounting for 90-95% of all diagnosed diabetes cases (1, 2). Worldwide, an estimated 537 million adults aged 20–79 years are currently living with diabetes (3, 4). The development of type 2 diabetes is mainly caused by two factors; impaired insulin secretion by pancreatic β-cells and the inability of insulin-sensitive tissues to respond to insulin (1, 5). Type 2 diabetes develops when the pancreas cannot produce enough insulin or when the insulin-sensitive tissues become resistant to insulin. Symptoms of T2D include excessive thirst and dry mouth, frequent urination, lack of energy, tiredness, slow healing wounds, recurrent infections in the skin, blurred vision, tingling or numbness in hands and feet (6). However, these symptoms can be mild or absent, making it possible for people with type 2 diabetes to live several years with the condition before being diagnosed.

Malaria is one of the most severe diseases of public health importance globally. With 247 million cases reported in 84 malaria endemic countries in 2021 (2), malaria remains a major cause of morbidity and mortality in many developing countries. The causative agent, the protozoan *Plasmodium*, malaria is transmitted to humans by infected female *Anopheles* mosquitoes. The human *Plasmodium* species include *Plasmodium vivax*, *Plasmodium ovale*, *Plasmodium malaria*, *Plasmodium falciparum* and *P. knowlesi* (2). The most dangerous of the plasmodia infecting humans is *P. falciparum* (7), and it is one of the most important causes of child mortality worldwide. The *P. falciparum* life cycle comprise a non-pathogenic symptomless extraerythrocytic stage, which is followed by the invasion of mature red blood cells by the infective forms (merozoites) and the initiation of pathogenic intraerythrocytic stages. Most of the clinical signs of malaria are caused by the parasite at stages in which it multiplies asexually in red blood cells. The most common clinical symptoms of severe malaria are high fever, progressing anemia, multi-organ dysfunction and unconsciousness or coma, which is one of the causes of death (7). The stages of the *P. falciparum* life cycle are complex, and this allows for the use of various evasion strategies by the malaria parasite. The immune response triggered against the malaria parasite is also complex and stage specific (8). Both the innate and adaptive immune responses are activated by the malaria parasite during malaria infection. The host immune responses play a key role in determining the efficiency of parasite clearance, resulting in immunological phenotype of individuals that are resistant or susceptible to malaria infection.

Several studies in recent times have reported the co-existence of malaria and type 2 diabetes in developing countries. However, most of the evidence reported in literature have been based on the effect of malaria on the risk of diabetes (9); the effect of diabetes on malaria outcome (10–12); and the effect of malaria on glycemic presentation (6). These findings have resulted in the emergence of hypotheses and pathways that have provided explanations for the co-existence of malaria and type 2 diabetes (13). The most popular of the hypotheses that has emerged to explain the co-existence of malaria and type 2 diabetes is the intra-uterine hypothesis. Summarily, the intra-uterine hypothesis is based on the effect of the malaria-induced inflammation which occurs during exposure to malaria *in utero* on the development of type 2 diabetes later in life.

The intra-uterine hypothesis arose as a plausible explanation for the increase in the burden of type 2 diabetes in malaria endemic populations. Low birth weight arising from of intra uterine stress has been linked with changes in skeletal muscle and pancreatic morphology function (14), leading to increased skeletal muscle insulin resistance and future risk of type 2 diabetes. Exposure to malaria *in utero* is associated with increased risk of preterm and low birthweight births (15) and may be a potential cause for later development of type 2 diabetes in life. A recent study reported subtle elevations of plasma glucose levels in young adult offspring of pregnancies affected by malaria which may be an early marker for the risk of later developing type 2 if these individuals are exposed to an obesogenic environment (16). Furthermore, malaria has been positively associated with insulin resistance (17) mediated by inflammation (18); which is an important risk factor for the development of type 2 diabetes (19). These findings give indirect evidence that inflammation is an important link for the co-existence of malaria and diabetes.

Cytokines are important mediators in the pathogenesis of malaria and T2D. Interestingly, the pro-inflammatory cytokines implicated in the pathogenesis of malaria such as IL-1β, IL-6, IL-8 and IL-12 (20, 21) have also been linked with the pathogenesis of T2D (22, 23). However, TNF- α is distinct among the main cytokines involved in the pathogenesis of malaria and T2D. TNF- α is a proinflammatory cytokine with pleiotropic properties (24). It is a multipotent cytokine produced by a wide range of immune cells such as B cells, T cells and macrophages. TNF- α is involved in various steps of immune responses and can initiate host defense, both innate and adaptive immune responses (25). TNF-α plays an important role in the pathogenesis of malaria (18, 26) and has also been implicated in the development of insulin resistance which contributes to the development of type 2 diabetes (27). In malaria infection, TNF- α is also involved in anti-Plasmodium responses that lead to intra-erythrocytic parasite killing and parasitemia reduction and it also plays a central role in the progression of malaria to cerebral malaria (21, 28). TNF-α upregulates the expression of adhesion receptors on endothelial cells, leading to an increase in the sequestration of parasite-infected RBCs on the endothelial cells in the brain. Furthermore, TNF-α is a major adipocyte cytokine; it interferes with insulin signaling after binding to its cognate receptor on muscle cells, thereby impairing insulin action (29–32). TNF-α has also been shown to increase leukocyte adhesion to endothelium, which results in endothelial dysfunction pathogenesis (33). *In vitro*, TNF-α has been shown to increase the transcriptional activity of TCF7L2 (a gene that has consistently been associated with type 2 diabetes) leading to reduced adipogenesis (34).

## Functional studies

TNF-α is an important human cytokine that is implicated in malaria pathogenicity (35). Malaria infected individuals produce varying levels of TNF-α; the level of TNF-α production is directly proportional to malaria severity (36). A high TNF production is associated with accelerated parasite clearance, while excessive TNF levels are associated with complications such as cerebral malaria or severe anemia (37). The pathway to TNF-α production in malaria infection begins at the pre-erythrocytic phase after the entry of *Plasmodium falciparum* parasite into the human host, sporozoite antigen stimulates the release of TNF-α before sporozoite invasion into the liver (38).

Following the release of merozoites from the liver, the merozoites infect red blood cells. In the red blood cells, molecular interactions occur between the merozoite and the red blood cell surface. Eventually, the merozoites develop into fully matured trophozoites which rupture to release merozoites, hemozoin and toxins such as *P. falciparum* glycosylphosphatidyl inositol into the bloodstream. This stimulates macrophages to produce TNF- α alongside other pro-inflammatory cytokines such as IFN-γ and IL-12 (39, 40), which coincides with the clinical manifestation of malaria. However, TNF- α is the main cytokine that has been associated with the severe forms of malaria, cerebral malaria (41–43).

Recent report shows that TNF-α plays dual role (which includes protective and pathogenic) in malaria physiopathology; as at higher levels, it promotes pathogenicity in cerebral malaria while it is protective against severe malaria at lower levels (44–46). This corroborates Clark et al. (47) findings, who reported that increased TNF- α production induces the cytoadherence of parasite-infected red blood cells to the endothelial cells lining blood vessels in the brain, a phenomenon known as sequestration which is characteristic of cerebral malaria.

Tumor necrosis factor (TNF)-α was the first proinflammatory cytokine to be implicated in the pathogenesis of insulin resistance and type 2 diabetes (48, 49). Several studies have demonstrated high levels of TNF- α in patients with type 2 diabetes. The major organs involved in the development of type 2 diabetes include the pancreas (β-cells and α-cells), liver, skeletal muscle, kidneys, brain, small intestine, and adipose tissue (50). TNF-α has been shown to reduce the expression of insulin-regulated glucose transporter type 4 (GLUT4), which is located mainly on adipocytes, and skeletal and cardiac muscles (48). The consequence of this is a reduction in insulin receptor substrate-1 (IRS-1) which results in impaired insulin sensitivity. Furthermore, TNF-α can act as an inhibitor of peripheral insulin action by inducing serine phosphorylation of insulin receptor substrate-1 which leads to insulin resistance (48).

Obesity is a major risk factor for type 2 diabetes (51). The level of mRNA of TNF-α and its protein has been shown to increase in the adipose tissues of diabetic individuals (52, 53). TNF-α impairs insulin sensitivity in adipose tissues is *via* the downregulation of protein level of insulin receptor substrate1 (IRS-1) and glucose transporter 4 (GLUT4) (53). The expression of TNF-α is increased in human adipose tissues with insulin resistance (27). In adipose tissues, TNF-alpha activates enhanced lipolysis leading to increased secretion of free fatty acids from adipose tissue into the circulation (49, 54). TNF-α mediates its biological effect in adipose tissues *via* the two receptors, TNFR1 and TNFR2. The TNFR1 is widely expressed on all cell types, while TNFR2 is expressed predominantly on leukocytes and endothelial cells. The two TNFRs have been shown to mediate distinct biological effects. The binding of TNF-α to TNFR1 mostly triggers pro-inflammatory pathways (55). The action of TNF-α on adipose tissues can also alter the production of many adipokines. This is relevant to the systemic effects of this TNF-α on insulin sensitivity and whole-body energy homeostasis (56). On the other hand, TNF-alpha upregulates the expression of genes like vascular cell adhesion molecule-1, plasminogen activator inhibitor-1, IL-6, IL-1b, angiotensinogen, resisting and leptin (57).

In obese individuals, TNF-α is over-expressed in adipose and muscle tissue (58). In obese type 2 diabetes patients, the TNF-*α* plasma level is related to the amount of visceral fat and is not instantly affected in poorly controlled diabetic patients by acute lowering of blood glucose level (59). Moreover, the levels of TNF-α expression strongly correlate with hyperinsulinemia and decreased insulin sensitivity (60). TNF-α also plays a vital role in the pathogenesis and development of obesity-induced insulin resistance as demonstrated by the augmented levels of TNF-α in systemic circulation, liver, and adipocytes (61–63). Impairment of normal functioning of the β-cells of pancreatic islets is one of the major causative factors for the suppression of insulin secretion. TNF-α can also induce the inflammation in pancreatic islets which lead to the induction of apoptosis in β-cells of pancreatic islets (64)

## TNF-α in genetic studies

Genetic variations in the promoter region of the TNF-α gene may regulate TNF- α production, transcription and affect susceptibility to or protection from inflammatory-related diseases such as malaria and type 2 diabetes (26, 65, 66). Many studies have been carried out to determine whether the TNF- α polymorphisms are associated with levels of TNF- α production, disease susceptibility and or disease severity (67–69). The polymorphisms at positions -238 (rs361525), -308 (rs1800629), -857 (rs1799724), -863 (rs1800630) and -1031(rs1799964) have been associated with increased transcriptional activity and production of TNF-α in several studies (20, 70). The polymorphism at the -308G/A has been linked with various inflammatory and autoimmune diseases (71–74). The -238 G/A polymorphism has been associated with insulin resistance syndrome and obesity (75). The polymorphisms at positions -857, -863 and -1031 have been associated with both increased luciferase activity and increased concanavalin-A stimulated TNF-α production from peripheral blood mononuclear cells (76). In malaria, the aforementioned SNPs have been associated with control of parasitemia levels and increased anti-*P. falciparum* IgG levels (77, 78); suggesting that the TNF- α SNPs may play a role in the effectiveness of anti-parasite responses. However, the control of TNF- α gene expression by these polymorphisms seems to be context-dependent based on secreting cell types, cell activation status, and action of other inflammatory mediators such as IL-6 and CRP (79).

A previous study by McGuire et al. (80) associated the A allele of TNF-α -308 with cerebral malaria. Gimenez et al. (36) also reported that TNF-α and TNF-α receptors are involved in the pathogenesis of cerebral malaria. Mohamedahmed and Abakar (81) implicated high TNF-α plasma levels with susceptibility to severe malaria, and this was corroborated with previous reports (26, 82, 83) that showed that TNF - 238G/A, a TNF-α SNP was associated with severe malaria and/or progression from uncomplicated malaria infection to severe malaria.

Single nucleotide variations in the TNF-α gene have also been implicated in increased insulin resistance typical of type 2 diabetes. The most widely studied of the TNF-α promoter variants are the *TNF-α* −308G/A and −238G/A polymorphisms. These TNF-α variants have been studied in association with the outcome of type 2 diabetes. Although several studies have focused on these associations, their conclusions have been controversial (84–86). An earlier meta-analysis on the association between *TNF-α* −308G/A and type 2 diabetes did not find any significant association (87). A more recent meta-analysis however, found the *TNF-α* −308G/A variant to be associated with susceptibility to type 2 diabetes (88). On other hand, the *TNF-α* −238G/A was not associated with type 2 diabetes in most of the previous meta-analyses carried out on the variant (87, 89). Generally, the association of 308G/A and 238G/A polymorphisms have been associated with insulin resistance, obesity and T2DM has been demonstrated in some ethnic groups (90–93).

## Discussion: TNF-α, an important link in the co-existence of malaria and type 2 diabetes

Malaria has long been associated with inflammation (94). Tumor necrosis factor (TNF)-α is an important pro-inflammatory cytokine involved in immune responses to malaria infection. It plays an important role in both the innate and adaptive immune responses to malaria parasites during malaria infection. Although inflammation is a major host defense mechanism against pathogens such as the *Plasmodium* parasite, inflammation can be harmful to the host with resultant acute or chronic pathology if dysregulated (20). As discussed earlier, TNF-α promotes pathogenicity in cerebral malaria at higher levels, while it is protective against severe malaria at lower levels (44–46).This is evident in malaria infection as increased serum levels of TNF-α is characteristic of malaria episodes and elevated levels correlates with faster parasite clearance with the resolution of malaria episodes (95, 96). On the other hand, increased TNF serum levels were repeatedly found in children with severe malaria (94, 97), establishing the important role that TNF-α plays in malaria pathogenesis. Interestingly, the TNF-α-induced inflammation seen in malaria infection is similar to that observed in type 2 diabetes (98). Studies have demonstrated high levels of TNF-α in patients with type 2 diabetes. TNF-α reduces the expression of insulin-regulated glucose transporter type 4 (48), resulting in impaired insulin sensitivity. Also, increased levels of TNF-α expression is strongly correlated with hyperinsulinemia and decreased insulin sensitivity (60).

The inflammation link between malaria and type 2 diabetes is not direct due to the complex nature of the inflammatory process in the two diseases. Nevertheless, previous studies have associated malaria-induced inflammation with critical risk factors of T2DM. A good example is the malaria-induced inflammation in placental malaria that has been associated with low birth weight, which is an important risk factor for the development of type 2 diabetes later in life (19, 99). Similarly, malaria has been positively associated with insulin resistance mediated by inflammation (17, 18), which is a key risk factor for the development of type 2 diabetes (19). One of the most important inflammatory mediators that has been implicated in the development of insulin resistance (which is central to the development of type 2 diabetes) is TNF-α which is also involved in the pathogenesis of malaria (18, 100). These observations affirm inflammation *via* TNF-α as a mechanism which contributes to the co-existence of malaria and type 2 diabetes in malaria endemic regions.

The pertinent question then, is, how does TNF-α connect malaria and type 2 diabetes? The answer lies in the well-known type 2 diabetes marker, TCF4 also known as TCF7L2 (transcription factor 7-like 2). The transcription factor 7-like 2 (TCF7L2) is the most potent locus for type 2 diabetes; as the association of *TCF7L2* with type 2 diabetes has been consistently replicated in multiple populations with diverse genetic origins (101). TNF-α has been shown to enhance the transcriptional activity of TCF7L2 *in vitro*, leading to reduced adipogenesis (54). The transcriptional effects of TNF-a promote oxidative stress and mitochondrial dysfunction, lipolysis and altered adipokine expression, thereby compromising insulin signaling and adipocyte lipid metabolism (56). Cawthorn et al. (54), found that TNF- α enhanced TCF4-dependent transcriptional activity during early anti-adipogenesis suggesting that TNF-α and Wnt signaling pathways may converge to inhibit adipocyte development at the level of TCF4-dependent gene transcription. TCF7L2 is a transcription factor that forms a basic part of the Wnt signaling pathway (101). This evidence further strengthens the possibility of a link between TNF-α and TCF7L2 which may play an important role in the co-existence of malaria and type 2 diabetes.

In order to test this hypothesis, we genotyped a TCF7L2 gene variant (rs7903146) in Nigerian children with malaria in a pilot study and found the gene to be present in the population. The study was carried out among under-five children in Ibadan, southwest Nigeria, an area that is holoendemic for malaria. The children were categorized into, asymptomatic, uncomplicated and severe malaria groups as defined by World Health Organization. Genotyping was done using PCR-RFLP, we found the TCF7L2 gene variant, rs7903146 in the population with a minor allelic frequency of 0.139. We also found the TCF7L2 variant, rs7903146 to be associated with susceptibility to developing severe malaria in children from Ibadan southwest Nigeria. These results suggest an association between the type 2 diabetes gene (TCF7L2) and malaria in Ibadan, Nigeria, a population that is malaria endemic.

**Figure 1:**
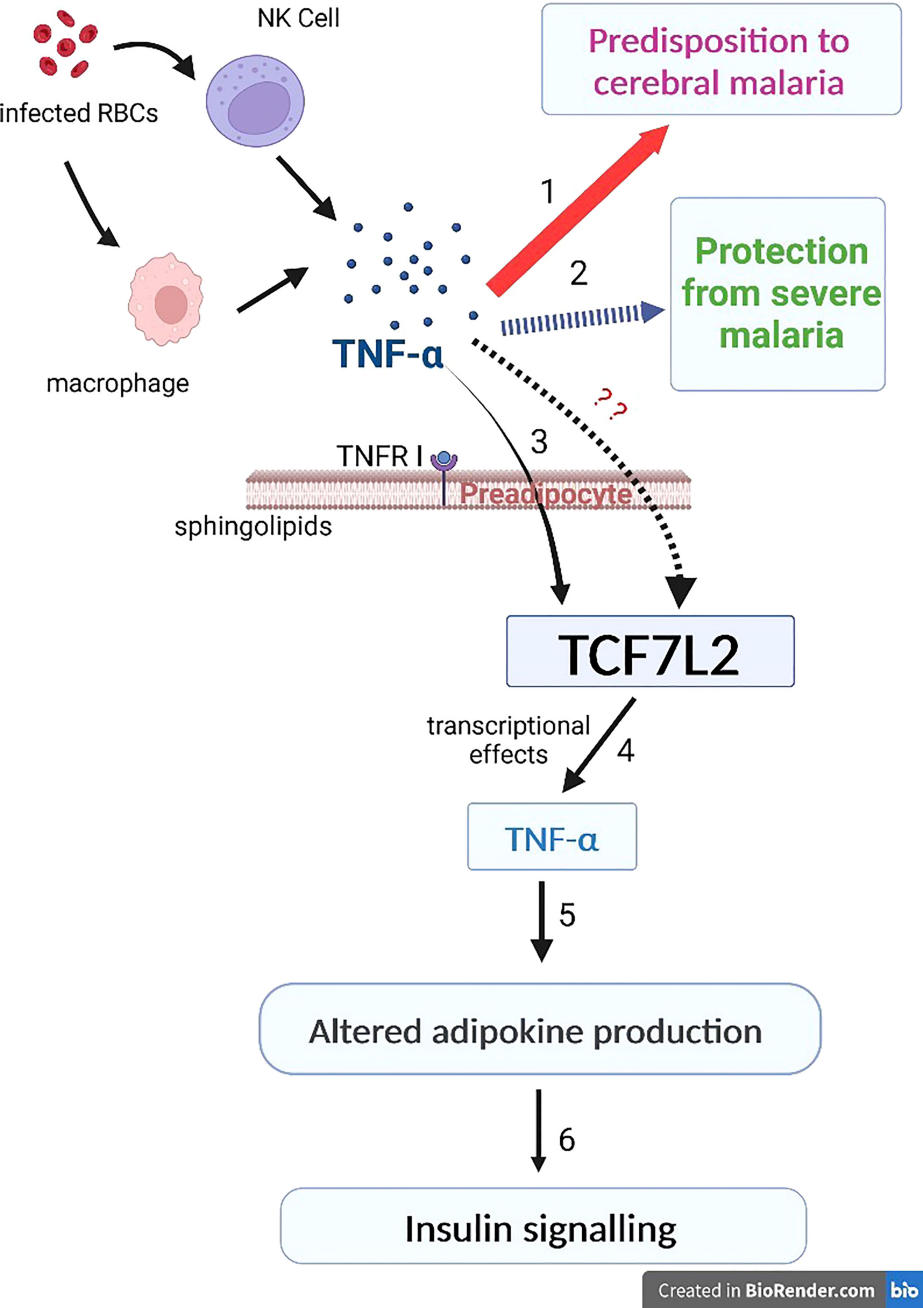
The inflammation link between malaria and type 2 diabetes. During malaria infection, infected erythrocytes lead to the activation of macrophages and natural killer cells which lead to the production of TNF-α. Varying levels of TNF-α determine the outcome of clinical malaria; the thick red arrow indicates high levels of TNF-α that promote cerebral malaria (1) while the dashed blue arrow indicates reduced levels of TNF-α that protects against severe malaria (2). 3-6: in type 2 diabetes, TNF-α mediates its biological effect in adipose tissues through its receptor, TNFR1. TNF-α binds to TNFR1, leading to the production of soluble forms of TNF-α. In preadipocytes, TNF-α can stimulate transcription via the type 2 diabetes gene, TCF7L2, leading to altered adipokine production, thereby compromising insulin signaling and adipocyte lipid metabolism (4-6; **Figure 1**). However, the effect(s) of varying levels of TNF-α on the transcriptional activity of TCF7L2 remains unknown (this is indicated by the red question marks).

This evidence suggests a probable pathway that links TNF-α and TCF7L2 in the co-existence of malaria and type 2 diabetes. During malaria infection, infected erythrocytes lead to the activation of macrophages and natural killer cells which lead to the production of TNF-α. However, varying levels of TNF-α determine the outcome of clinical malaria; high levels of TNF-α promote cerebral malaria while reduced levels of TNF-α protects against severe malaria (1-3; **Figure 1**). In type 2 diabetes, TNF-α can mediate its biological effect in adipose tissues through its receptor, TNFR1. TNF-α binds to its receptor, TNFR1, leading to the production of soluble forms of TNF-α. In preadipocytes, TNF-α can stimulate transcription *via* the type 2 diabetes gene, TCF7L2, leading to altered adipokine production, thereby compromising insulin signaling and adipocyte lipid metabolism (4-6; **Figure 1**). However, the effect(s) of varying levels of TNF-α on the transcriptional activity of TCF7L2 remains unknown (7; **Figure 1**). Studies are needed to investigate the effect of varying levels of TNF-α on the transcription of TCF7L2.

There are critical knowledge gaps which requires further studies. One of these is the effect of the varying levels of TNF-α on the transcriptional activity of TCF7L2. This is important because TNFα is known to have differential effects depending on its level of production *in vivo*. However, the effect(s) of its elevated or reduced levels on the transcriptional activity of the type 2 diabetes gene, TCF7L2 remains unknown. Another knowledge gap is the dearth of studies on malaria and type 2 diabetes in children and adolescents. Most of the research that has been carried out on the co-existence of malaria with diabetes has been conducted using the adult population with little or no studies on children. This is important mainly because malaria is most severe in children and the incidence of type 2 diabetes is becoming more common in children and adolescents.

## Data availability statement

The original contributions presented in the study are included in the article/Supplementary Material. Further inquiries can be directed to the corresponding author.

## Ethics statement

The studies involving human participants were reviewed and approved by Oyo State Ministry of Health Ethics Review Board. Written informed consent to participate in this study was provided by the participants’ legal guardian/next of kin.

## Author contributions

SA and OA conceived and designed the idea behind the paper, OB analyzed the data and participated in the manuscript drafting and revision. All authors contributed to the article and approved the submitted version.

## References

[1] IDF. International diabetes federation diabetes atlas (2021). Available at: https://diabetesatlas.org/resources/.

[2] WHO. Diabetes. fact sheets. World Health Organization (2022). Available at: https://www.who.int/news-room/fact-sheets/detail/diabetes.

[3] Haamid B Majid S Khan M.S., K.S. Bhat MH Hamid R Ashraf R. Impact of pro- and anti- inflammatory biomarkers on development and severity of type 2 diabetes mellitus – a case-control study. Heliyon (2022) 8(11):e11329. doi: 10.2139/ssrn.4071641 36387548 PMC9641208

[4] International Diabetes Federation. International diabetes federation atlas(ninth edition) (2019). Available at: www.diabetesatlas.org.

[5] Roden M Shulman GI. The integrative biology of type 2 diabetes. Nature (2019) 576:51–60. doi: 10.1038/s41586-019-1797-8 31802013

[6] Metta E Bailey A Kessy F Geubbels E Hutter I Haisma H. In a situation of rescuing life. In: Meanings given to diabetes symptoms and care-seeking practices among adults in southeastern Tanzania: a qualitative inquiry, vol. 15. BMC Public Health (2015). doi: 10.1186/s12889-015-1504-0 PMC4358854 25886626

[7] Trampuz A Jereb M Muzlovic I Prabh MJ. Clinical review: severe malaria. Crit Care (2003) 7.4:315–23. doi: 10.1186/cc2183 PMC270697 12930555

[8] Malaguarnera L Musumeci S. The immune response to *Plasmodium falciparum* malaria. Lancet Infect Dis (2002) 2:472–8. doi: 10.1016/S1473-3099(02)00344-4 12150846

[9] Kalra S Khandelwal D Singla R Aggarwal S Dutta D. Malaria and diabetes. J Of Pakistan Med Assoc (2017) 67(5):810–3. 28507380

[10] Danquah I Bedu-Addo G Mockenhaupt FP. Type 2 diabetes mellitus and increased risk for malaria infection. Emerg Infect Dis (2010) 16(10):1601–4. doi: 10.3201/eid1610.100399 PMC3294394 20875289

[11] Ikekpeazu EJ Ikekpeazu EJ Neboh EE Maduka IC Nwagbara IJ Nwobodo MW. Type-2 diabetes mellitus and malaria parasitaemia: effect on liver function tests. Asian J Med Sci (2010) 2(5):214–7.

[12] Carrillo-Larco RM Altez-Fernandez C Ugarte-Gil C. Is diabetes associated with malaria and malaria severity? a systematic review of observational studies [version 3; peer review: 2 approved]. Wellcome Open Res (2019) 4. doi: 10.12688/wellcomeopenres.15467.1 PMC6961413 31976376

[13] Ch’Ng JH Moll K Wyss K Hammar U Rydén M Kämpe O. Enhanced virulence of plasmodium falciparum in blood of diabetic patients. PloS One (2021) 16(6 June). doi: 10.1371/journal.pone.0249666 PMC8211161 34138868

[14] Christensen DL Kapur A Bygbjerg IC. Physiological adaption to maternal malaria and other adverse exposure: low birth weight, functional capacity, and possible metabolic disease in adult life. Int J Gynecol Obstet (2011) 115(SUPPL. 1). doi: 10.1016/S0020-7292(11)60006-4 22099434

[15] Thompson JM Eick SM Dailey C Dale AP Mehta M Nair A. Relationship between pregnancy-associated malaria and adverse pregnancy outcomes: a systematic review and meta-analysis. J Trop Pediatr (2020) 66(3):327–38. doi: 10.1093/TROPEJ/FMZ068 31598714

[16] Grunnet LG Bygbjerg IC Mutabingwa TK Lajeunesse-Trempe F Nielsen J Schmiegelow C. Influence of placental and peripheral malaria exposure in fetal life on cardiometabolic traits in adult offspring. BMJ Open Diabetes Res Care (2022) 10(2). doi: 10.1136/bmjdrc-2021-002639 PMC8981354 35379692

[17] Acquah S Boampong JN Jnr BAE. Functional paradox of leptin and adiponectin in diabetes patients and controls in the cape coast metropolis of Ghana. Med J Dr DY Patil Univ (2017) 10(3):268. doi: 10.4103/MJDRDYPU.MJDRDYPU_271_16

[18] Acquah S Boampong JN Jnr E Ackon B. Increased oxidative stress and inflammation independent of body adiposity in diabetic and nondiabetic controls in falciparum malaria. BioMed Res Int (2016) 2016:5216913. doi: 10.1155/2016/5216913 PMC4889793 27298824

[19] Lontchi-Yimagou E Sobngwi E Matsha TE Kengne AP. Diabetes mellitus and inflammation. Curr Diabetes Rep (2013) 13(3):435–44. doi: 10.1007/s11892-013-0375-y 23494755

[20] Penha-Gonçalves C. Genetics of malaria inflammatory responses: a pathogenesis perspective. Front Immunol (2019) 10:1771. doi: 10.3389/fimmu.2019.01771 31417551 PMC6682681

[21] Popa GL Popa MI. Recent advances in understanding the inflammatory response in malaria: a review of the dual role of cytokines. J Immunol Res (2021) 2021:1–9. doi: 10.1155/2021/7785180 PMC8592744 34790829

[22] Alexandraki K Piperi C Kalofoutis C Singh J Alaveras A Kalofoutis A. Inflammatory process in type 2 diabetes: the role of cytokines. Ann N Y Acad Sci (2006) 1084:89–117. doi: 10.1196/annals.1372.039 17151295

[23] Liu C Feng X Li Q Wang Y Li Q Hua M. Adiponectin, TNF-α and inflammatory cytokines and risk of type 2 diabetes: a systematic review and meta-analysis. Cytokine (2016) 86:100–9. doi: 10.1016/j.cyto.2016.06.028 27498215

[24] Horiuchi T Mitoma H Harashima SI Tsukamoto H Shimoda T. Transmembrane TNF-α: structure, function and interaction with anti-TNF agents. Rheumatology (Oxford) (2010) 49(7):1215–28. doi: 10.1093/rheumatology/keq031 PMC2886310 20194223

[25] Horiuchi T Mitoma H Harashima S Tsukamoto H Shimoda T. Review transmembrane TNF- a: structure, function and interaction with anti-TNF agents. (2010) :1215–28. doi: 10.1093/rheumatology/keq031 PMC2886310 20194223

[26] Olaniyan SA Amodu OK Bakare AA Troye-Blomberg M Omotade OO Rockett KA. Tumour necrosis factor alpha promoter polymorphism, TNF-238 is associated with severe clinical outcome of falciparum malaria in ibadan southwest Nigeria. Acta Tropica (2016) 161(2015):62–7. doi: 10.1016/j.actatropica.2016.05.006 27178813

[27] Peraldi P Hotamisligil GS Buurman WA White MF Spiegelman BM. Tumor necrosis factor (TNF)-β inhibits insulin signaling through stimulation of the p55 TNF receptor and activation of sphingomyelinase. J Biol Chem (1996) 271(22):13018–22. doi: 10.1074/jbc.271.22.13018 8662983

[28] Clark IA Virelizier JL Carswell EA Wood PR. Possible importance of macrophage-derived mediators in acute malaria. Infect Immun (1981) 32:1058–66. doi: 10.1128/iai.32.3.1058-1066.1981 PMC351558 6166564

[29] Hotamisligil GS Arner P Caro JF Atkinson RL Spiegelman BM. Increased adipose tissue expression of tumor necrosis factor-alpha in human obesity and insulin resistance. J Clin Invest (1995) 95(5):2409–15. doi: 10.1172/JCI117936 PMC295872 7738205

[30] Peraldi P Spiegelman B. TNF-alpha and insulin resistance: summary and future prospects. Mol Cell Biochem (1998) 182(1-2):169–75. doi: 10.1023/A:1006865715292 9609126

[31] Ruan H Lodish HF. Insulin resistance in adipose tissue: direct and indirect effects of tumor necrosis factor alpha. Cytokine Growth Factor Rev (2003) 14:447–55. doi: 10.1016/S1359-6101(03)00052-2 12948526

[32] Dandona P Aljada A Chaudhuri A Mohanty P Garg R. Metabolic syndrome: a comprehensive perspective based on interactions between obesity, diabetes, and inflammation. Circulation (2005) 111(11):1448–54. doi: 10.1161/01.CIR.0000158483.13093.9D 15781756

[33] Zeng M Zhang H Lowell C He P. Tumor necrosis factor-alpha-induced leukocyte adhesion and microvessel permeability. Am J Physiol Heart Circ Physiol (2002) 283(6):H2420–30. doi: 10.1152/ajpheart.00787.2001 12388263

[34] Kabagambe EK Glasser SP Ordovas JM Warodomwichit D Tsai MY Hopkins PN. TCF7L2 polymorphisms and inflammatory markers before and after treatment with fenofibrate. Diabetol Metab Syndrome (2009) 1(1). doi: 10.1186/1758-5996-1-16 PMC2766367 19825152

[35] Randall LM Engwerda CR. Experimental parasitology TNF family members and malaria: old observations, new insights and future directions. Exp Parasitol (2010) 126(3):326–31. doi: 10.1016/j.exppara.2010.04.016 20433831

[36] Gimenez F Lagerie B Fernandez C Pino P Mazier D. Tumor necrosis factor a in the pathogenesis of cerebral. Cell Mol Life Sci (2003) 60:1623–35. doi: 10.1007/s00018-003-2347 PMC11138823 14504653

[37] Grau GE Taylor TE Molyneux ME. Tumour necrosis factor and disease severity in children with falciparum malaria. New Engl J Med (1989) 320:1586–91. 10.1056/NEJM198906153202404 2657427

[38] Richards AL. Tumour necrosis factor and associated cytokines in the host’s response to malaria. Int J Parasitol (1997) 27:1251–63. doi: 10.1016/S0020-7519(97)00122-7 9394195

[39] Stevenson MM Riley EM. Innate immunity to malaria. Nat Rev Immunol (2004) 4(3):169–80. doi: 10.1038/nri1311 15039754

[40] Kwiatkowski D Perlman P. Harwood Academic Publishers (1999).

[41] Storm J Craig AG. Pathogenesis of cerebral malaria–inflammation and cytoadherence. Front Cell Infect Microbiol (2014) 29;4:100. doi: 10.3389/fcimb.2014.00100 PMC4114466 25120958

[42] Mauduit M Depinay N Franetich JF Gru AC Chavatte M Luty AJF. Inhibitory effect of TNF- a on malaria pre-erythrocytic stage Development: influence of host Hepatocyte/Parasite combinations. PLoS One (2011) 6(3):e17464. doi: 10.1371/journal.pone.0017464 21394207 PMC3048870

[43] Perera MK Herath NP Pathirana SL Alles HK Mendis KN Premawansa S. Association of high plasma TNF-alpha levels and TNF-alpha/IL-10 ratios with TNF2 allele in severe p. falciparum malaria patients in Sri Lanka. Pathog Glob Health (2013) 107(1):21–9. doi: 10.1179/2047773212Y.0000000069 PMC4001599 23432860

[44] Pino P Mazier D. Tumor necrosis factor a in the pathogenesis of cerebral. Cell Mol Life Sci September (2003). doi: 10.1007/s00018-003-2347-x PMC11138823 14504653

[45] Awasthi G Singh S Dash AP Das A. Genetic characterization and evolutionary inference of TNF- α through computational analysis. Braz J Infect Dis (2008) 12:374–9. doi: 10.1590/S1413-86702008000500006 19219275

[46] Leão L Puty B Dolabela MF Povoa MM Gecy Y Né DS. Association of cerebral malaria and TNF- α levels: a systematic review. BMC Infect Dis (2020) 20(1):442. doi: 10.1186/s12879-020-05107-2 32576141 PMC7310527

[47] Clark IA Alleva LM Mills AC Cowden WB. Pathogenesis of malaria and clinically similar conditions. Clin Microbiol Rev (2004) 17:509–39. doi: 10.1128/CMR.17.3.509-539.2004 PMC452556 15258091

[48] Hotamisligil GS. Inflammation and endoplasmic reticulum stress in obesity and diabetes. Int J Obes (2009) 32(S7):S52–4. doi: 10.1038/ijo.2008.238 PMC2885768 19136991

[49] Sethi JK. The role of TNF in adipocyte metabolism. Semin Cell Dev Biol (1999) 10:19–29. doi: 10.1006/scdb.1998.0273 10355025

[50] Defronzo RA. From the triumvirate to the ominous octet: a new paradigm for the treatment of type 2 diabetes mellitus. Diabetes (2009) 58:773–95. doi: 10.2337/db09-9028 PMC2661582 19336687

[51] Galicia-Garcia U Benito-Vicente A Jebari S Larrea-Sebal A Siddiqi H Uribe KB. Pathophysiology of type 2 diabetes mellitus. Int J Mol Sci (2020) 21(17):1–34. doi: 10.3390/ijms21176275 PMC7503727 32872570

[52] Akash MSH Rehman K Liaqat A. Tumor necrosis factor-alpha: role in development of insulin resistance and pathogenesis of type 2 diabetes mellitus. J Cell Biochem (2018) 119(1):105–10. doi: 10.1002/jcb.26174 28569437

[53] el Hini SH Ahmed ATZ Hamed EMS Mahmoud YZ Eldin AMK Abdelghany HM. Pivotal role of both tnf-α 238g/a and tcf7l2 c/t gene polymorphisms in type 2 diabetes. Open Access Macedonian J Med Sci (2020) 8(F):283–6. doi: 10.3889/oamjms.2020.5008

[54] Cawthorn WP Heyd F Hegyi K Sethi JK. Tumour necrosis factor-α inhibits adipogenesis *via* a β-catenin/TCF4(TCF7L2)-dependent pathway. Cell Death Differentiation (2007) 14(7):1361–73. doi: 10.1038/sj.cdd.4402127 PMC4303765 17464333

[55] Yang S Wang J Brand DD Zheng SG. Role of TNF-TNF receptor 2 signal in regulatory T cells and its therapeutic implications. Front Immunol (2018) 19(9):784. doi: 10.3389/fimmu.2018.00784 PMC5916970 29725328

[56] Cawthorn WP Sethi JK. TNF-α and adipocyte biology. FEBS Lett (2008) 582(1):117–31. doi: 10.1016/j.febslet.2007.11.051 PMC4304634 18037376

[57] Saghizadeh M Ong JM Garvey WT Henry RR Kern PA. The expression of TNF by human muscle relationship to insulin resistance. in. J Clin Invest (1996) 97(4). doi: 10.1172/JCI118504 PMC507159 8613535

[58] Hoffmann C Lorenz K Braithwaite SS Colca JR Palazuk BJ Hotamisligil GS. Altered gene expression for tumor necrosis factor-alpha and its receptors during drug and dietary modulation of insulin resistance. Endocrinology (1994) 134:264–70. doi: 10.1210/endo.134.1.8275942 8275942

[59] Alzamil H. Elevated serum TNF-*α* is related to obesity in type 2 diabetes mellitus and is associated with glycemic control and insulin resistance. J Obese (2020) 2020:5076858. doi: 10.1155/2020/5076858 PMC7013317 32089876

[60] Sethi JK Hotamisligil GS. Metabolic messengers: tumour necrosis factor. Nat Metab (2021) 3(10):1302–12. doi: 10.1038/s42255-021-00470-z 34650277

[61] Kabayama K Sato T Kitamura F Uemura S Kang BW Igarashi Y. TNFα-induced insulin resistance in adipocytes as a membrane microdomain disorder: involvement of ganglioside GM3. Glycobiology (2005) 15:21–9. doi: 10.1093/glycob/cwh135 15306563

[62] Solomon S Odunusi O Carrigan D Majumdar G Kakoola D Lenchik N. TNF-α inhibits insulin action in liver and adipose tissue: a model of metabolic syndrome. Hormone Metab Res (2010) 42:115–21. doi: 10.1055/s-0029-1241834 19960405

[63] da Rocha AF Liboni TF Kurauti MA de Souza CO Miksza DR Moreira CCL. Tumor necrosis factor alpha abolished the suppressive effect of insulin on hepatic glucose production and glycogenolysis stimulated by cAMP. Pharmacol Rep (2014) 66:380–5. doi: 10.1016/j.pharep.2013.12.005 24905512

[64] Wang C Guan Y Yang J. Cytokines in the progression of pancreatic β-cell dysfunction. Int J Endocrinol (2010) 2010:515136. doi: 10.1155/2010/515136 21113299 PMC2989452

[65] Jamil K Jayaraman A Ahmad J Joshi S Kumar YS. TNF-alpha -308G/A and – 238G/A polymorphisms and its protein network associated with type 2 diabetes mellitus. Saudi J Biol Sci (2017) 24:1195–203. doi: 10.1016/j.sjbs.2016.05.012 PMC5562469 28855812

[66] Mahto H Rina T Meher BR Prusty BK Sharma M Deogharia D. TNF- α promoter polymorphisms (G-238A and G-308A) are associated with susceptibility to systemic lupus erythematosus (SLE) and p. falciparum malaria: a study in malaria endemic area. Sci Rep (2019) 9(1):11752. doi: 10.1038/s41598-019-48182-5 31409832 PMC6692415

[67] De Jong BA Westendorp AM Bakker AM Huizinga M. Polymorphisms in or near tumour necrosis factor gene do not determine levels of endotoxin-induced TNF production. Genes Immun (2002) 3:25–9. 10.1038/sj.gene.6363824 11857057

[68] Feng R Li Y Zhao D Wang C Niu Y. Lack of association between TNF 238 G/A polymorphism and type 2 diabetes: a meta-analysis. Acta Diabetol (2009) 46:339–43. doi: 10.1007/s00592-009-0118-3 19367363

[69] Sharma N Joseph R Arun R Chandni R Srinivas KL Banerjee M. Cytokine gene polymorphism (interleukin-1β +3954, interleukin-6 [-597/-174] and tumor necrosis factor-a - 308) in chronic periodontitis with and without type 2 diabetes mellitus. Indian J Dent Res (2014) 25:375–80. doi: 10.4103/0970-9290.138343 25098998

[70] Wilson AG Symons JA McDowell TL McDevitt HO Duff GW. Effects of a polymorphism in the human tumor necrosis factor alpha promoter on transcriptional activation. Proc Natl Acad Sci USA (1997) 94:3195–9. doi: 10.1073/pnas.94.7.3195 PMC20345 9096369

[71] Verjans GM Brinkman BNM Van Doornik CEM. Polymorphism of tumour necrosis factor-alpha (TNF-α) at position -308 in relation to ankylosing spondylitis. Clin Exp Immunol (1994) 97:45–7. 10.1111/j.1365-2249.1994.tb06577.x PMC1534773 8033419

[72] Cabrera M Shaw MA Sharples C. Polymorphism in tumour necrosis factor genes associated with mucocutaneous leishmaniasis. J Exp Med (1995) 182:1259–64. doi: 10.1084/jem.182.5.1259 PMC2192198 7595196

[73] Wilson AG Clay FE Crane AM. Comparative genetic association of human leukocyte antigen class II and tumour necrosis factor alpha with dermatitis herpetiformis. J Invest Dermatol (1995) 104:856–8. 10.1111/1523-1747.ep12607031 7738367

[74] Nadel S Newport MJ Booy R. Variation in the tumour necrosis factor-alpha gene promoter region may be associated with death from meningococcal disease. J Infect Dis (1996) 174:878–80. doi: 10.1093/infdis/174.4.878 8843235

[75] Rasmussen SK Urhammer SA Jensen JN. The -238 and -308 G/A polymorphisms of tumour necrosis factor alpha gene promoter are not associated with features of the insulin resistance syndrome or altered birth weight in Danish caucasians. J Clin Endocrinol Metab (2000) 85:1731–4. 10.1210/jcem.85.4.6563 10770222

[76] Higuchi T Seki N Kamizono S Yamada A Kimura A Kato H. Polymorphism of the 5’-flanking region of the human tumor necrosis factor (TNF)-alpha gene in japanese. *Tissue antigens* . 51 (1998), 605–12. 10.1111/j.1399-0039.1998.tb03002.x 9694352

[77] Wilson AG Symons JA McDowell TL McDevitt HO Duff GW. Effects of a polymorphism in the human tumor necrosis factor alpha promoter on transcriptional activation. Proc Natl Acad Sci USA (1997) 94:3195–9. 10.1073/pnas.94.7.3195 PMC20345 9096369

[78] Basu M Maji AK Chakraborty A Banerjee R Mullick S Saha P. Genetic association of toll-like-receptor 4 and tumor necrosis factor-a polymorphisms with plasmodium falciparum blood infection levels. Infect Genet Evol (2010) 10:686–96. doi: 10.1016/j.meegid.2010.03.008 20307689

[79] El-Tahan RR Ghoneim AM El-Mashad N. TNF-a gene polymorphisms and expression. Springerplus (2016) 5:1508. doi: 10.1186/s40064-016-3197-y 27652081 PMC5014780

[80] McGuire W Hill AV Allsopp CE Greenwood BM Kwiatkowski D. Variation in the TNF-alpha promoter region associated with susceptibility to cerebral malaria. *Nature* . 371 (1994), 508–10. doi: 10.1038/371508a0 7935762

[81] Mohamedahmed KA Abakar A. The role of TNF- α levels as predictive diagnostic biomarker among children with severe falciparum malaria in endemic area in Sudan. International Journal of Academic Health and Medical Research (IJAHMR) (2019) 3(7):1–6.

[82] Flori L Delahaye NF Iraqi FA Fumoux F Rihet P. TNF as a malaria candidate gene: polymorphism-screening and family-based association analysis of mild malaria attack and parasitemia in Burkina Faso. Genes Immun (2005) 6(6):472–80. doi: 10.1038/sj.gene.6364231 15931230

[83] Randall LM Kenangalem E Lampah DA Tjitra E Mwaikambo ED Handojo T. A study of the TNF/LTA/LTB locus and susceptibility to severe malaria in highland papuan children and adults. Malar J (2010) 9:302. doi: 10.1186/1475-2875-9-302 21029472 PMC2978234

[84] Kim EY Priatel JJ Teh SJ The HS. TNF receptor type 2 (p75) functions as a costimulator for antigen-driven T cell responses *in vivo* . J Immunol (2006) 176(2):1026–35. doi: 10.4049/jimmunol.176.2.1026 16393990

[85] Liu C Batliwalla F Li W. Genome-wide association scan identifies candidate polymorphisms associated with differential response to anti-TNF treatment in rheumatoid arthritis. Mol Med (2008) 14:575–81. doi: 10.2119/2008-00056 PMC2276142 18615156

[86] Golshani Y Zarei M Mohammadi S. Acute/Chronic pain relief: is althaea officinalis essential oil effective? Avicenna J Neuro Psych Physiol (2015) 2(4). doi: 10.17795/ajnpp-36586

[87] Feng R-N Zhao C Sun C-H Li Y. Meta-analysis of *TNF* 308 G/A polymorphism and type 2 diabetes mellitus. PloS One (2011) 6(4):e18480. doi: 10.1371/journal.pone.0018480 21494616 PMC3072982

[88] Guo Y Ren M Ge L Sun C Li R Ma C. Increased serum concentrations of TNF-like weak inducer of apoptosis predict higher 28-day mortality in patients with sepsis. Emergency Med Int (2019) 2019:1–7. doi: 10.1155/2019/7238705 PMC6348793 30733876

[89] Gao W Zhu R Yang L. Association of tumor necrosis factor-Alpha-308 G/A and -238 G/A polymorphism with diabetic retinopathy: a systematic review and updated meta-analysis. Ophthalmic Res (2021) 64(6):903–15. doi: 10.1159/000513586 PMC8743915 33279899

[90] Guzma´ n-Flores JM Escalante M Sa´nchez-Corona J Garcıa-Zapie´n AG Cruz Quevedo EG Mun˜ oz-Valle JF. Association analysis between -308G/A and -238G/A TNF-alpha gene promoter polymorphisms and insulin resistance in Mexican women with gestational diabetes mellitus. J Invest Med (2013) 61:265–9. doi: 10.2310/JIM.0b013e31827b98c9 23254337

[91] Saxena M Srivastava N Banerjee M. Association of IL-6, TNF-a and IL 10 gene polymorphisms with type 2 diabetes mellitus. Mol Biol Rep (2013) 40:6271–9. doi: 10.1007/s11033-013-2739-4 24057184

[92] Sikka R Raina P Matharoo K Bandesh K Bhatia R Chakrabarti S. TNF-a (g.-308 g> a) and ADIPOQ (g. +45 T >G) gene polymorphisms in type 2 diabetes and microvascular complications in the region of punjab (North-West India). Curr Eye Res (2014) 39:1042–51. doi: 10.3109/02713683.2014.892998 24655058

[93] Wilson AG di Giovine FS Blakemore AIF Duff GW. Single base polymorphism in the human tumor necrosis factor alpha (TNF-alpha) gene detectable by NcoI restriction of PCR product. Hum Mol Genet (1992) 1:353. 10.1093/hmg/1.5.353 1363876

[94] Kwiatkowski D Hill AV Sambou I Twumasi P Castracane J Manogue KR. TNF concentration in fatal cerebral, non-fatal cerebral, and uncomplicated plasmodium falciparum malaria. Lancet (1990) 336(8725):1201–4. doi: 10.1016/0140-6736(90)92827-5 1978068

[95] Clark K Nyambati VCS Neitz AWH Louw AI. In search of an amino acid falciparum. Biochem Afr - 2nd FASBMB; 15th SASBMB (1998).

[96] McGuire W D’Alessandro U Stephens S Olaleye BO Langerock P Greenwood BM. Levels of tumour necrosis factor and soluble TNF receptors during malaria fever episodes in the community. Trans R Soc Trop Med Hygiene (1998) 92:50–3. doi: 10.1016/S0035-9203(98)90951-8 9692151

[97] Tchinda VH Tako EA Tene G Fogako J Nyonglema P. Severe malaria in cameroonian children: correlation between plasma levels of three soluble inducible adhesion molecules and TNF-alpha. Acta Trop (2007) 102:20–8. doi: 10.1016/j.actatropica.2007.02.011 17397790

[98] Acquah S. Linking malaria to type 2 diabetes mellitus: a review. J Ghana Sci Assoc (2019) 18(1):56–70.

[99] Fitri LE Sardjono TW Rahmah Z Siswanto B Handono K Dachlan YP. Low fetal weight is directly caused by sequestration of parasites and indirectly by IL-17 and IL-10 imbalance in the placenta of pregnant mice with malaria, (2015). Korean J Parasitol (2015) 53(2):189–96. doi: 10.3347/kjp.2015.53.2.189 PMC4416375 25925177

[100] Nasr A Allam G Hamid O. IFN-gamma and TNF associated with severe falciparum malaria infection in Saudi pregnant women. Malar J (2014) 13:314. doi: 10.1186/1475-2875-13-314 25124540 PMC4137072

[101] Del Bosque-Plata L Martínez-Martínez E Espinoza-Camacho MÁ Gragnoli C. The role of *TCF7L2* in type 2 diabetes. Diabetes (2021) 70(6):1220–8. doi: 10.2337/db20-0573 PMC8275893 34016596

